# Supplementary material for: HIV as a Host Susceptibility State for Severe Drug Hypersensitivity: Disentangling Biological Susceptibility from Drug Exposure in the FAERS Database
Source: medRxiv. 2026 Jul 9:2026.07.07.26356279. Preprint. [Version 1] doi: 10.64898/2026.07.07.26356279 (PMC13370540; doi:10.64898/2026.07.07.26356279)
Supplement: Supplementary file 1 [file NIHPP2026.07.07.26356279v1-supplement-1.pdf]

medRxiv preprint doi: https://doi.org/10.64898/2026.07.07.26356279; this version posted July 9, 2026. The copyright holder for this preprint (which was not certified by peer review) is the author/funder, who has granted medRxiv a license to display the preprint in perpetuity.
It is made available under a CC-BY 4.0 International license .

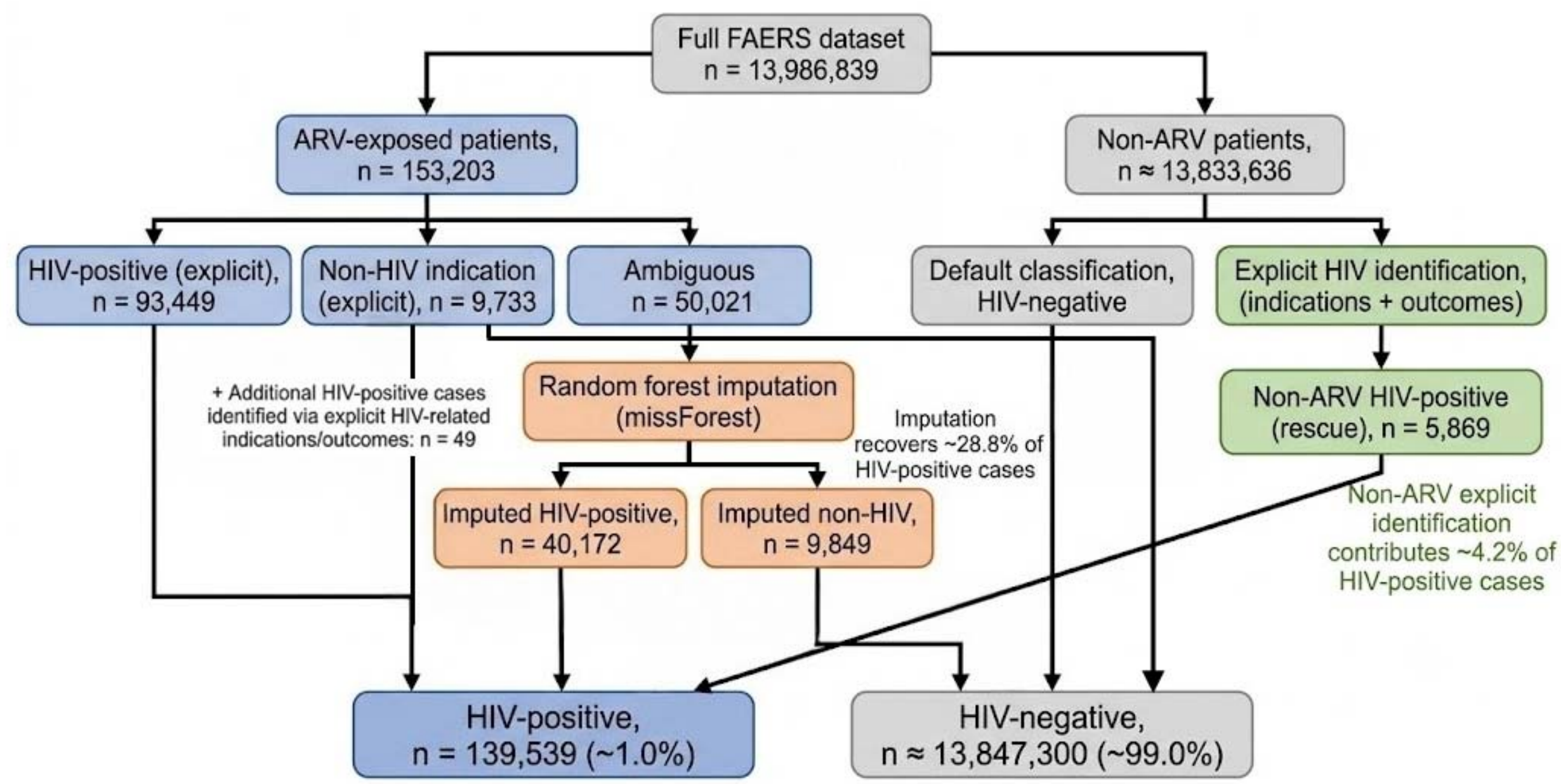

HIV Flag	N	pct
ARV+ explicit	93,449	66.97%
ARV+ imputed	40,172	28.79%
Non-ARV explicit	5,869	4.21%
ARV+ rescue	49	0.04%

Figure S1. HIV status classification and imputation pipeline. Hierarchical workflow used to assign HIV status within the FAERS database. Among antiretroviral (ARV)-exposed reports, individuals with explicit HIV-related indications were classified as HIV-positive, whereas those with explicit non-HIV indications were classified as HIV-negative. Reports with ambiguous indications underwent random forest imputation using demographic characteristics, geographic region, medication burden, clinical outcomes, antiretroviral exposures, and drug principal components. Among non-ARV-exposed reports, HIV-positive cases were identified through explicit HIV-related indications or outcomes. The random forest model demonstrated excellent out-of-bag performance (proportion falsely classified [PFC] = 0.00097), indicating minimal classification error. Application of this framework yielded 139,539 HIV-positive reports (approximately 1.0% of all FAERS reports), including 40,172 HIV-positive reports identified through imputation and 5,869 additional HIV-positive reports identified among non-ARV-exposed individuals.

medRxiv preprint doi: https://doi.org/10.64898/2026.07.07.26356279; this version posted July 9, 2026. The copyright holder for this preprint
(which was not certified by peer review) is the author/funder, who has granted medRxiv a license to display the preprint in perpetuity.
It is made available under a CC-BY 4.0 International license .

medRxiv preprint doi: https://doi.org/10.64898/2026.07.07.26356279; this version posted July 9, 2026. The copyright holder for this preprint (which was not certified by peer review) is the author/funder, who has granted medRxiv a license to display the preprint in perpetuity.
It is made available under a CC-BY 4.0 International license .

Group	drug_name	Total SCAR	SJS-TEN	DRESS	AGEP	GBFDE
TOTAL	Any Drug	930	458	425	67	1
NRTI	NRTI TOTAL	251	131	118	5	0
NNRTI	NNRTI TOTAL	221	178	39	4	0
PI	PI TOTAL	73	21	48	10	0
INSTI	INSTI TOTAL	91	35	57	2	0
Booster	Booster TOTAL	8	4	5	0	0
Non-ARV	Non-ARV TOTAL	286	89	158	46	1
NRTI	lamivudine	85	60	25	0	0
NRTI	abacavir	62	19	44	2	0
NRTI	emtricitabine	47	18	27	2	0
NRTI	tenofovir_disoproxil	33	20	12	1	0
NRTI	zidovudine	17	10	7	0	0
NRTI	tenofovir	4	2	2	0	0
NRTI	tenofovir_alafenamide	3	2	1	0	0
NNRTI	nevirapine	175	157	17	1	0
NNRTI	efavirenz	32	15	14	3	0
NNRTI	rilpivirine	8	3	5	0	0
NNRTI	etravirine	4	3	1	0	0
NNRTI	doravirine	2	0	2	0	0
PI	ritonavir	32	8	25	2	0
PI	darunavir	28	8	16	7	0
PI	lopinavir	7	0	6	1	0
PI	atazanavir	3	2	1	0	0
PI	fosamprenavir	3	3	0	0	0
INSTI	raltegravir	48	11	39	1	0
INSTI	dolutegravir	28	14	14	0	0
INSTI	bictegravir	13	8	4	1	0
INSTI	cabotegravir	1	1	0	0	0
INSTI	elvitegravir	1	1	0	0	0
Booster	cobicistat	8	4	5	0	0
Non-ARV	hydroxychloroquine	29	1	6	22	0
Non-ARV	trimethoprim	25	14	11	0	0
Non-ARV	sulfamethoxazole	24	13	11	0	0
Non-ARV	azithromycin	12	0	3	9	0
Non-ARV	ribavirin	11	4	4	3	0
Non-ARV	rifampin	9	0	9	0	0
Non-ARV	clarithromycin	7	4	6	0	0
Non-ARV	furosemide	7	0	7	0	0
Non-ARV	levetiracetam	7	1	6	0	0
Non-ARV	acetaminophen	6	6	0	1	1
Non-ARV	isoniazid	6	2	4	0	0
Non-ARV	levofloxacin	6	0	6	0	0
Non-ARV	carbamazepine	5	5	0	0	0
Non-ARV	enoxaparin	5	1	2	2	0

medRxiv preprint doi: https://doi.org/10.64898/2026.07.07.26356279; this version posted July 9, 2026. The copyright holder for this preprint (which was not certified by peer review) is the author/funder, who has granted medRxiv a license to display the preprint in perpetuity.
It is made available under a CC-BY 4.0 International license .

Non-ARV	lansoprazole	5	0	5	0	0
Non-ARV	dapsone	4	1	3	0	0
Non-ARV	omeprazole	4	0	4	0	0
Non-ARV	piperacillin	4	0	4	0	0
Non-ARV	rifabutin	4	0	4	0	0
Non-ARV	tazobactam	4	0	4	0	0
Non-ARV	telaprevir	4	1	2	1	0
Non-ARV	allopurinol	3	2	1	0	0
Non-ARV	esomeprazole	3	0	3	0	0
Non-ARV	peginterferon_alfa-2a	3	1	3	0	0
Non-ARV	pyrazinamide	3	0	3	0	0
Non-ARV	valproate	3	1	2	0	0
Non-ARV	vancomycin	3	1	2	0	0
Non-ARV	anakinra	2	0	2	0	0
Non-ARV	boceprevir	2	0	2	0	0
Non-ARV	clindamycin	2	0	1	1	0
Non-ARV	dalteparin	2	2	0	0	0
Non-ARV	hydroxyzine	2	0	2	0	0
Non-ARV	lamotrigine	2	1	1	0	0
Non-ARV	leucovorin	2	0	2	0	0
Non-ARV	lorazepam	2	0	2	0	0
Non-ARV	methylprednisolone	2	1	1	0	0
Non-ARV	oseltamivir	2	1	1	0	0
Non-ARV	phenytoin	2	2	0	0	0
Non-ARV	pregabalin	2	1	2	0	0
Non-ARV	sarilumab	2	0	2	0	0
Non-ARV	sulfasalazine	2	0	2	0	0
Non-ARV	acetazolamide	1	0	1	0	0
Non-ARV	acetylcysteine	1	0	1	0	0
Non-ARV	acyclovir	1	1	0	0	0
Non-ARV	amiodarone	1	0	1	0	0
Non-ARV	amlodipine	1	0	0	1	0
Non-ARV	amoxicillin	1	0	1	0	0
Non-ARV	aspirin	1	1	0	0	0
Non-ARV	bupropion	1	1	0	0	0
Non-ARV	cefixime	1	1	0	0	0
Non-ARV	ceftriaxone	1	0	0	1	0
Non-ARV	chloroquine	1	0	0	1	0
Non-ARV	ciprofloxacin	1	0	1	0	0
Non-ARV	cisplatin	1	0	0	1	0
Non-ARV	clofazimine	1	0	1	0	0
Non-ARV	clonazepam	1	1	0	0	0
Non-ARV	clonidine	1	0	1	0	0
Non-ARV	clopidogrel	1	0	1	0	0
Non-ARV	clozapine	1	0	1	0	0

medRxiv preprint doi: https://doi.org/10.64898/2026.07.07.26356279; this version posted July 9, 2026. The copyright holder for this preprint (which was not certified by peer review) is the author/funder, who has granted medRxiv a license to display the preprint in perpetuity.
It is made available under a CC-BY 4.0 International license .

Non-ARV	colchicine	1	1	0	0	0
Non-ARV	daptomycin	1	0	1	0	0
Non-ARV	elbasvir	1	1	0	0	0
Non-ARV	ethambutol	1	0	1	0	0
Non-ARV	fluconazole	1	1	0	0	0
Non-ARV	gabapentin	1	1	0	0	0
Non-ARV	interferon_alfa-2a	1	0	0	1	0
Non-ARV	iopamidol	1	0	1	0	0
Non-ARV	iopromide	1	0	1	0	0
Non-ARV	itraconazole	1	1	1	0	0
Non-ARV	ivermectin	1	1	0	0	0
Non-ARV	ketoconazole	1	1	0	0	0
Non-ARV	lacosamide	1	1	0	0	0
Non-ARV	loperamide	1	1	0	0	0
Non-ARV	lumefantrine	1	1	0	0	0
Non-ARV	methotrexate	1	1	0	0	0
Non-ARV	metronidazole	1	1	0	0	0
Non-ARV	nitrofurantoin	1	0	1	0	0
Non-ARV	nystatin	1	0	1	0	0
Non-ARV	ondansetron	1	0	0	1	0
Non-ARV	oxycodone	1	1	0	0	0
Non-ARV	pantoprazole	1	0	1	0	0
Non-ARV	paroxetine	1	1	0	0	0
Non-ARV	pentoxifylline	1	0	1	0	0
Non-ARV	prednisolone	1	0	0	1	0
Non-ARV	prednisone	1	1	0	0	0
Non-ARV	risperidone	1	0	1	0	0
Non-ARV	rituximab	1	1	0	0	0
Non-ARV	testosterone	1	1	0	0	0
Non-ARV	thiamine	1	0	1	0	0
Non-ARV	tigecycline	1	0	1	0	0
Non-ARV	ustekinumab	1	1	0	0	0
Non-ARV	valacyclovir	1	0	1	0	0
Non-ARV	valganciclovir	1	0	1	0	0

Table S2. Primary-Suspect Drugs in SCAR among PLWH.

medRxiv preprint doi: https://doi.org/10.64898/2026.07.07.26356279; this version posted July 9, 2026. The copyright holder for this preprint (which was not certified by peer review) is the author/funder, who has granted medRxiv a license to display the preprint in perpetuity.
It is made available under a CC-BY 4.0 International license .

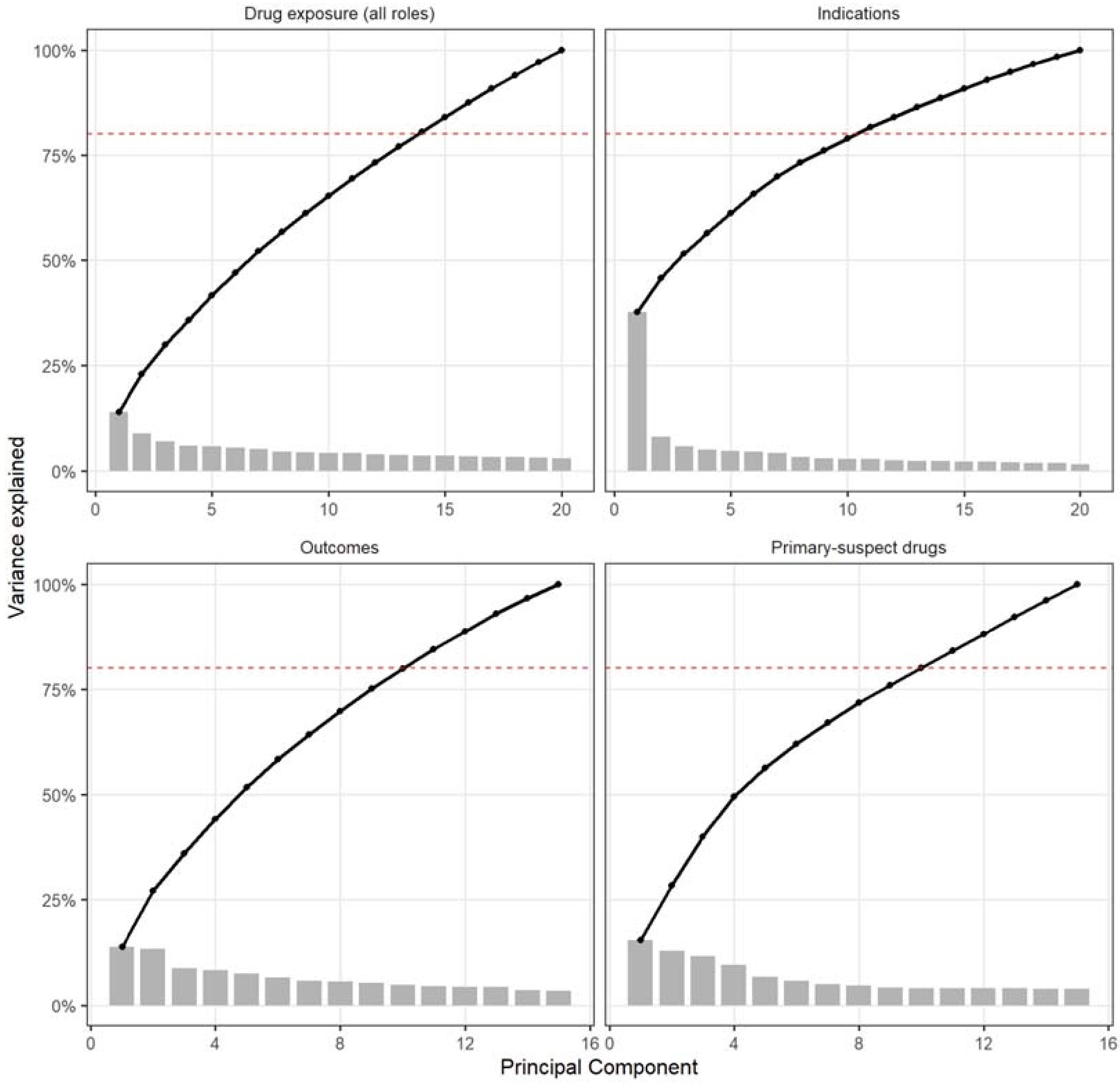

Figure S3. Principal component structure of drug exposures, indications, outcomes, and primary-suspect medications. Scree plots demonstrating the proportion of variance explained by principal components derived from four high-dimensional feature matrices: all reported drug exposures, indications, adverse event outcomes, and primary-suspect drugs. The dashed red line denotes 80% cumulative variance explained. Across all feature sets, variance was concentrated within a limited number of principal components, with approximately 8–14 components capturing 80% of total variance.

medRxiv preprint doi: https://doi.org/10.64898/2026.07.07.26356279; this version posted July 9, 2026. The copyright holder for this preprint (which was not certified by peer review) is the author/funder, who has granted medRxiv a license to display the preprint in perpetuity.
It is made available under a CC-BY 4.0 International license .

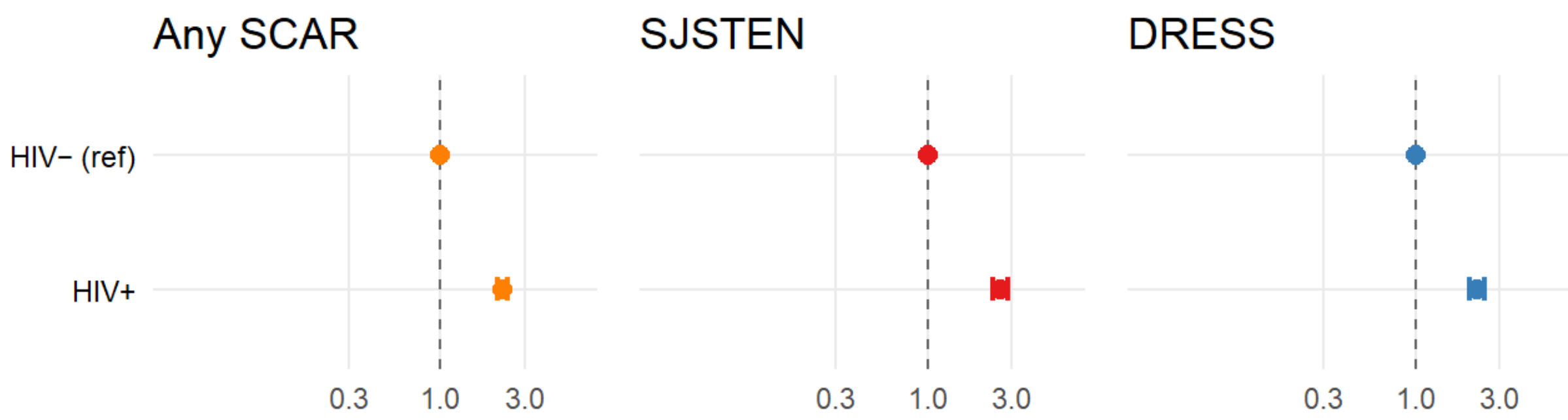

Figure S4. Unadjusted association between HIV infection and severe cutaneous adverse reactions. HIV infection was associated with increased odds of any SCAR (OR 2.26, 95% CI [2.11-2.41]), SJS/TEN (OR 2.57 [2.33-2.82]), and DRESS (OR 2.23 [2.46-2.82]). Estimates represent crude associations without adjustment for demographic characteristics, reporting patterns, medication burden, or drug exposure structure.

medRxiv preprint doi: https://doi.org/10.64898/2026.07.07.26356279; this version posted July 9, 2026. The copyright holder for this preprint (which was not certified by peer review) is the author/funder, who has granted medRxiv a license to display the preprint in perpetuity.
It is made available under a CC-BY 4.0 International license .

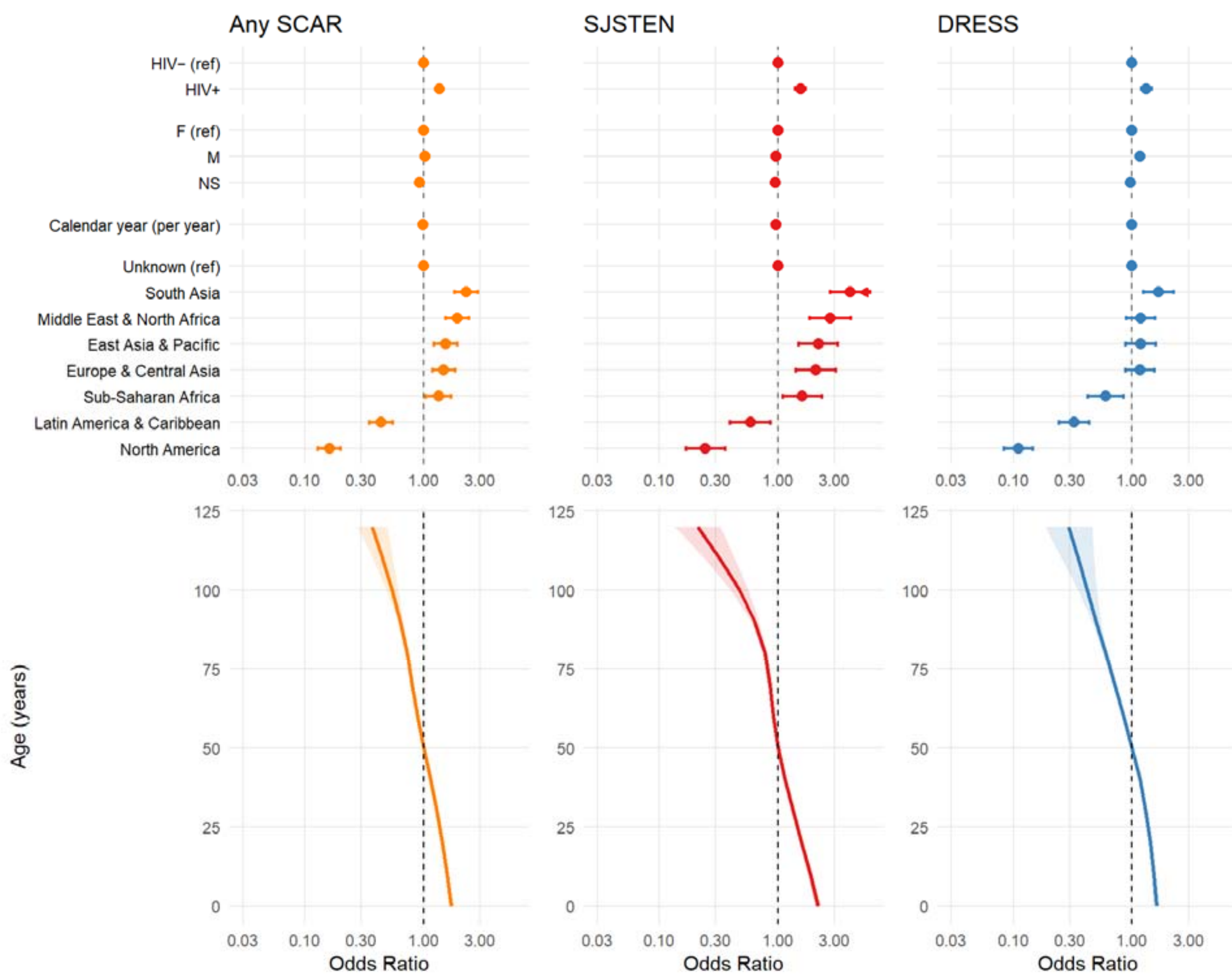

Figure S5. Demographic-adjusted association between HIV infection and severe cutaneous adverse reactions. Models were adjusted for age (natural cubic spline with 4 degrees of freedom), sex, geographic region, and calendar year. HIV infection remained associated with increased odds of all three SCAR outcomes – any SCAR (OR 1.37, 95% CI [1.28-1.47]), SJS/TEN (OR 1.55 [1.41-1.71]), and DRESS (OR 1.32 [1.20-1.46]) – after demographic adjustment, although effect estimates were attenuated relative to unadjusted analyses.

medRxiv preprint doi: https://doi.org/10.64898/2026.07.07.26356279; this version posted July 9, 2026. The copyright holder for this preprint (which was not certified by peer review) is the author/funder, who has granted medRxiv a license to display the preprint in perpetuity.
It is made available under a CC-BY 4.0 International license .

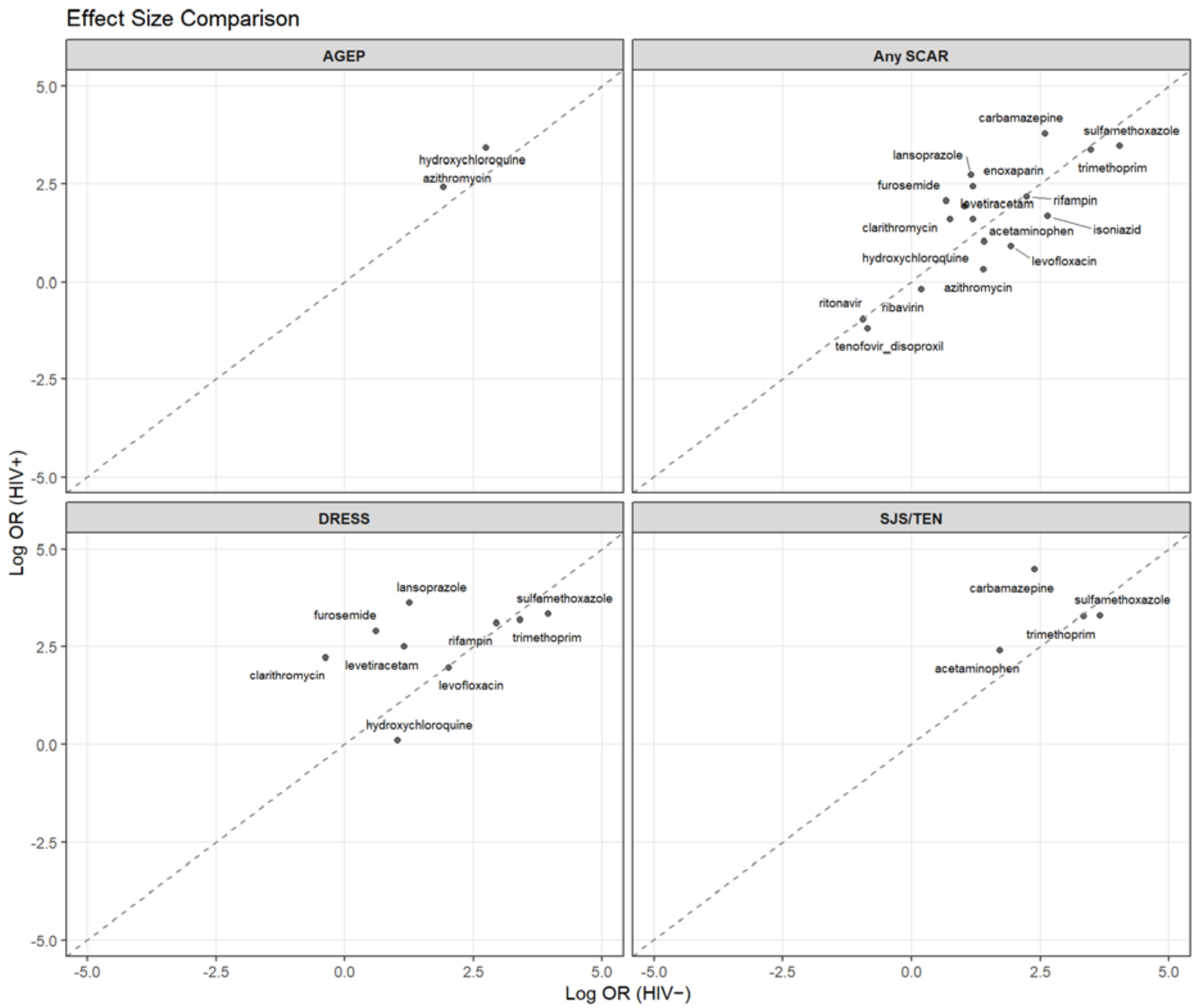

Figure S6. Comparison of drug-associated effect sizes in HIV-positive and HIV-negative individuals. Scatterplots comparing drug-specific RORs in HIV-negative (x-axis) and HIV-positive (y-axis) individuals for AGEP, any SCAR, DRESS, and SJS/TEN. Each point represents a primary suspect drug. The dashed diagonal line indicates equal effect sizes across HIV strata. Most drugs clustered near the line of identity, indicating similar effect sizes in HIV-positive and HIV-negative individuals, while a subset of drugs demonstrated stronger associations among HIV-positive individuals, particularly for DRESS and SJS/TEN.

medRxiv preprint doi: https://doi.org/10.64898/2026.07.07.26356279; this version posted July 9, 2026. The copyright holder for this preprint (which was not certified by peer review) is the author/funder, who has granted medRxiv a license to display the preprint in perpetuity.
It is made available under a CC-BY 4.0 International license .

Outcome	Causative Drug	#Reports	#Reports w/ROR025 Positive Comed	% w/ROR025 Positive Comed	Median #ROR025 Positive Comeds	Top 5 ROR025 Positive Comeds
SCAR	TMP-SMX	25	14	56%	1	nevirapine (n=9, 36%); lamivudine (n=4, 16%); ethambutol (n=2, 8%); isoniazid (n=2, 8%); pyrazinamide (n=2, 8%)
SCAR	carbamazepine	5	5	100%	2	TMP-SMX (n=5, 100%); lamivudine (n=5, 100%)
SCAR	clarithromycin	7	7	100%	8	abacavir (n=7, 100%); raltegravir (n=6, 85.7%); ethambutol (n=4, 57.1%); fluconazole (n=4, 57.1%); rifabutin (n=4, 57.1%)
SCAR	rifampin	9	9	100%	5	raltegravir (n=9, 100%); ethambutol (n=7, 77.8%); isoniazid (n=7, 77.8%); pyrazinamide (n=7, 77.8%); rifabutin (n=3, 33.3%)
SCAR	hydroxychloroquine	29	29	100%	3	ritonavir (n=28, 96.6%); darunavir (n=20, 69%); azithromycin (n=10, 34.5%); ceftriaxone (n=6, 20.7%); lopinavir (n=5, 17.2%)
SCAR	levetiracetam	7	7	100%	4	ritonavir (n=7, 100%); darunavir (n=6, 85.7%); abacavir (n=4, 57.1%); clobazam (n=2, 28.6%); ethambutol (n=2, 28.6%)
SCAR	lansoprazole	5	5	100%	2	perindopril (n=5,

medRxiv preprint doi: https://doi.org/10.64898/2026.07.07.26356279; this version posted July 9, 2026. The copyright holder for this preprint (which was not certified by peer review) is the author/funder, who has granted medRxiv a license to display the preprint in perpetuity.
It is made available under a CC-BY 4.0 International license .

						100%); raltegravir (n=5, 100%)
SCAR	furosemide	7	7	100%	12	enoxaparin (n=7, 100%); hydroxychloroquine (n=7, 100%); hydroxyzine (n=7, 100%); lopinavir (n=7, 100%); ritonavir (n=7, 100%)
SJSTEN	TMP-SMX	14	12	85.70%	1	nevirapine (n=8, 57.1%); lamivudine (n=4, 28.6%); ethambutol (n=2, 14.3%); isoniazid (n=2, 14.3%); pyrazinamide (n=2, 14.3%)
SJSTEN	carbamazepine	5	5	100%	2	TMP-SMX (n=5, 100%); lamivudine (n=5, 100%)
SJSTEN	clarithromycin	4	4	100%	13	abacavir (n=4, 100%); fluconazole (n=4, 100%); TMP-SMX (n=3, 75%); acyclovir (n=3, 75%); atorvastatin (n=3, 75%)
SJSTEN	hydroxychloroquine	1	1	100%	1	abacavir (n=1, 100%)
SJSTEN	levetiracetam	1	1	100%	7	abacavir (n=1, 100%); clobazam (n=1, 100%); ethambutol (n=1, 100%); metronidazole (n=1, 100%); moxifloxacin (n=1, 100%)
DRESS	TMP-SMX	11	2	18.20%	0	fluconazole (n=1, 9.1%); nevirapine (n=1, 9.1%)
DRESS	clarithromycin	6	6	100%	10.5	abacavir (n=6, 100%); raltegravir (n=6, 100%); TMP-SMX (n=3,

medRxiv preprint doi: https://doi.org/10.64898/2026.07.07.26356279; this version posted July 9, 2026. The copyright holder for this preprint (which was not certified by peer review) is the author/funder, who has granted medRxiv a license to display the preprint in perpetuity.
It is made available under a CC-BY 4.0 International license .

						50%); acyclovir (n=3, 50%); allopurinol (n=3, 50%)
DRESS	Rifampin	9	9	100%	5	raltegravir (n=9, 100%); ethambutol (n=7, 77.8%); isoniazid (n=7, 77.8%); pyrazinamide (n=7, 77.8%); rifabutin (n=3, 33.3%)
DRESS	hydroxychloroquine	6	6	100%	3	ritonavir (n=6, 100%); enoxaparin (n=4, 66.7%); lopinavir (n=3, 50%); azithromycin (n=2, 33.3%); ceftriaxone (n=2, 33.3%)
DRESS	levetiracetam	6	6	100%	4	darunavir (n=6, 100%); ritonavir (n=6, 100%); abacavir (n=3, 50%); leucovorin (n=2, 33.3%); TMP-SMX (n=1, 16.7%)
DRESS	lansoprazole	5	5	100%	2	perindopril (n=5, 100%); raltegravir (n=5, 100%)
DRESS	furosemide	7	7	100%	12	enoxaparin (n=7, 100%); hydroxychloroquine (n=7, 100%); hydroxyzine (n=7, 100%); lopinavir (n=7, 100%); ritonavir (n=7, 100%)
AGEP	hydroxychloroquine	22	22	100%	2	ritonavir (n=22, 100%); darunavir (n=20, 90.9%); azithromycin (n=8, 36.4%); ceftriaxone (n=4, 18.2%); lopinavir (n=2, 9.1%)

medRxiv preprint doi: https://doi.org/10.64898/2026.07.07.26356279; this version posted July 9, 2026. The copyright holder for this preprint (which was not certified by peer review) is the author/funder, who has granted medRxiv a license to display the preprint in perpetuity.
It is made available under a CC-BY 4.0 International license .

Table S7. Co-medication burden among HIV-positive SCAR reports involving selected non-antiretroviral primary suspect drugs
